# Genetic diversity of *Escherichia coli* isolated from ice cube production sites

**DOI:** 10.1186/s13104-018-3737-3

**Published:** 2018-09-10

**Authors:** Novi Pusparini, Diana E. Waturangi, Tepy Usia, Eva Nikastri

**Affiliations:** 1 Faculty of Biotechnology, Atma Jaya Catholic University of Indonesia, Jalan Jenderal Sudirman 51, Jakarta, 12930 Indonesia; 2 National Agency of Drug and Food Control, Jalan Percetakan Negara 23, Jakarta, 10560 Indonesia; 3 Research Center of Drug and Food, National Agency for Drug and Food Control, Jalan Percetakan Negara 23, Jakarta, 10560 Indonesia

**Keywords:** *E. coli*, Genetic diversity, ERIC-PCR, REP-PCR, Ice cube

## Abstract

**Objective:**

The prevalence of *Escherichia coli* including from ice cubes in Indonesia is quite high. Unfortunately, little is known about the genetic diversity of *E. coli* from ice cube production site. Genotypic variation in *E. coli* populations is a major barrier to control public health risk associated with foodborne pathogen. The aims of this study were to analyze the genotypic diversity of *E. coli* strains isolated from various samples in order to determine the genetic relationship between those strains. This study is also important to understand the occurrence, prevalence and profile picture of different pathogenic *E. coli* in various sources which potentially cause disease.

**Results:**

Enterobacterial repetitive intergenic consensus (ERIC) and repetitive extragenic palindromic polymerase chain reaction (REP-PCR) dendrogram showed high genetic diversity of 120 *E. coli* isolates in majority of sampling sites. DNA fingerprint patterns showed 26 and 21 clusters with 11 and 3 fingerprints individual lineages for ERIC and REP-PCR respectively. There was no correlation observed between phylogenetic relationship and virulence genes. The result indicated a variation of *E. coli* isolates in ice cube manufacturers. ERIC-PCR method is more discriminative compared with REP-PCR to analyze the genetic diversity of *E. coli* from ice cubes production sites.

## Introduction

It was reported by WHO in 2015 that diarrhoeal diseases are responsible for more than half of the global burden of foodborne diseases, causing 550 million people to fall ill and 230,000 deaths every year. Diarrhoea is often caused by eating raw or undercooked meat, eggs, fresh produce, dairy products as well as drinking water contaminated by norovirus, *Campylobacter*, non-typhoidal *Salmonella* and pathogenic *Escherichia coli* (*E. coli*) [1].

Iced beverage are commonly consumed in Indonesia mostly produced by industries or homemade, nonetheless it may not always prepared properly. Several previous studies have revealed the presence of foodborne pathogenic bacteria in the ice and iced beverages, such as Salmonella as *Salmonella* spp, *E. coli* especially pathogenic *E. coli* and *Vibrio cholerae* [2–4]. These conditions indicate the potency of microbiological hazard in iced beverages production. The microbiological hazard may come from the ingredients of iced beverages or from the processing environment.

Currently in Indonesia, little is known about the prevalence of different pathogenic *E. coli* in various sources including from ice cubes. Therefore, it is essential to generate a prior knowledge about the prevalence of pathogens especially *E. coli* in different source. Understanding their occurrence also the genetic diversity analysis in various potential host sources of contamination would help us to assess the health risk posed by such strains of *E. coli* and epidemiological surveillance of bacterial infections [5]. High genetic diversity among *E. coli* populations potentially influence the accuracy of the results, thus, more information is necessary on the genetic diversity of *E. coli* populations in a host source of interest.

Regarding the method of typing microbial pathogens or identifying bacteria at the strain level, ERIC and REP-PCR typing had the ability to separate *Lactobacillus* strains in based upon the profiles generated [6, 7]. Our results also indicated that the method is applicable to high throughput analysis. High genetic diversity also presented by ERIC-PCR results for *E. coli* in dairies products [8]. Both the ERIC and REP-PCR were shown high discriminatory index in *Salmonella enteritidis* research by Fardsanei et al. [9]. Thus, we use those methods in this research.

The aims of this study were to analyze the genotypic diversity of *E. coli* strains isolated from various samples using REP and ERIC-PCR in order to determine the genetic relationship between those strains. This study is also important to understand the occurrence and prevalence of different pathogenic *E. coli* in various sources. The data could be used to determine *E. coli* profile picture which potentially cause disease in Indonesia.

## Main text

### Methods

Materials used in this research was bacterial genomic DNA of positive *E. coli* isolates collected from ice cube manufacturers in previous study [10]. Ice cube manufacturers were located in city A, city B and city C from all big cities in Indonesia as representative. One hundred and twenty-one samples consists of 63 samples from city A, 50 samples from city B and 8 samples from city C. Study sites were collected from several area in Indonesia. Six sampling points at ice cube manufacturers were taken from water as raw material for ice, water filtration results, rinse water from hands of workers, rinse water from container where ice is released from the mold, rinse water from hook tool and ice cubes as final products.

*Escherichia coli* was cultured in *E. coli* Broth (Oxoid) and EMB Agar (Oxoid) was used as selective media. Incubation temperature for *E. coli* is 37 °C for 24 h. Positive *E. coli* colonies were picked randomly 4 for each plate. Bacterial genomic DNA was extracted from all *E. coli* isolates. A loopful of bacteria colonies in 500 μL sterile water were prepared for each sample. DNA was extracted using Wizard genomic DNA purification kit (Promega) according to the manufacturer’s instructions. Before it was used, the DNA samples were quantified using BioDrop-DUO (Isogen Life Science). DNA were stored at − 20 °C until used.

Genotyping was performed using ERIC-PCR and REP-PCR fingerprinting assay. The PCR amplifications were performed in a volume 25 μL of reaction mixtures contained 12.5 μL Go Taq (Promega); 1.25 μL of 10 ρmol ERIC1R (5′-ATGTAAGCT CCTGGGGAT-3′) and ERIC2 (5′-AAGTAAGTGACTGGGGGTGAGC-3′) [11]; 1.5 μL of DNA template; 8.5 μL of PCR grade water for ERIC-PCR and 12.5 μL of Go Taq (Promega, USA); 1.5 μL of 10 ρmol REP 1R (5′-III ICGICGICA TCIGGC-3′) and REP 2I (5′-ICGICTTATCIGGCCTAC-3′) [12]; 2 μL of DNA template; and 7.5 μL of PCR grade water for REP-PCR. The ERIC-PCR and REP-PCR thermal cycler (Biometra Tpersonal) program for this method followed Adiguzel et al. [11], since the other journals that has been tried does not give better band appearance. Amplified PCR products were separated by electrophoresis on 1.0% (w/v) agarose (1st base) at 60 V for 3 h. The molecular size of fragments generated by electrophoresis were determined by comparison to 1-kb DNA ladders (Gene aid).

To study the relatedness of bacterial strains, banding patterns generated by ERIC and REP-PCR was scored using binary scoring system that recorded the presence and absence of bands as 1 and 0, respectively. To produce dendrogram, a binary matrix was analyzed using dice similarity coefficient and unweighted pair group method for arithmetic averages (UPGMA) [7, 13]. These analysis were carried out using Free Tree software. UPGMA dendrogram was drawn using Treeview [14–16].

### Results

**Fig. 1:**
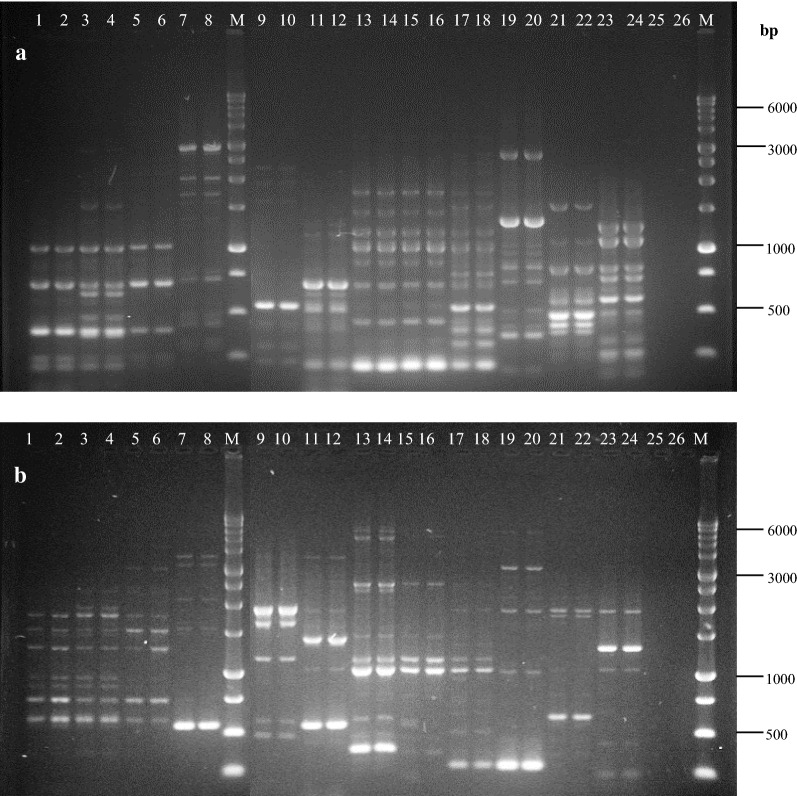
DNA fingerprinting of several isolates *E. coli* generated by ERIC-PCR (**a**) and REP-PCR (**b**) amplification. *M* marker 1 kb ladder (gene aid)

A total of 121 *E. coli* isolates from ice cube manufacturers were analyzed in this study. The genomic DNA concentration isolated from these samples were ranged between 50 and 1500 ng/µL with A260/A80 ratio between 1.9 and 2.0. DNA samples concentration tested were standardized to 100 ng/µL. The sizes of ERIC-PCR products ranged from about 150 to about 4000 bp while the sizes of REP-PCR products ranged from about 170 to about 4000 bp (Fig. 1). DNA fingerprint of ERIC-PCR in this study obtained 7–14 bands, where REP-PCR profiles resulted 5–10 bands.

**Fig. 2:**
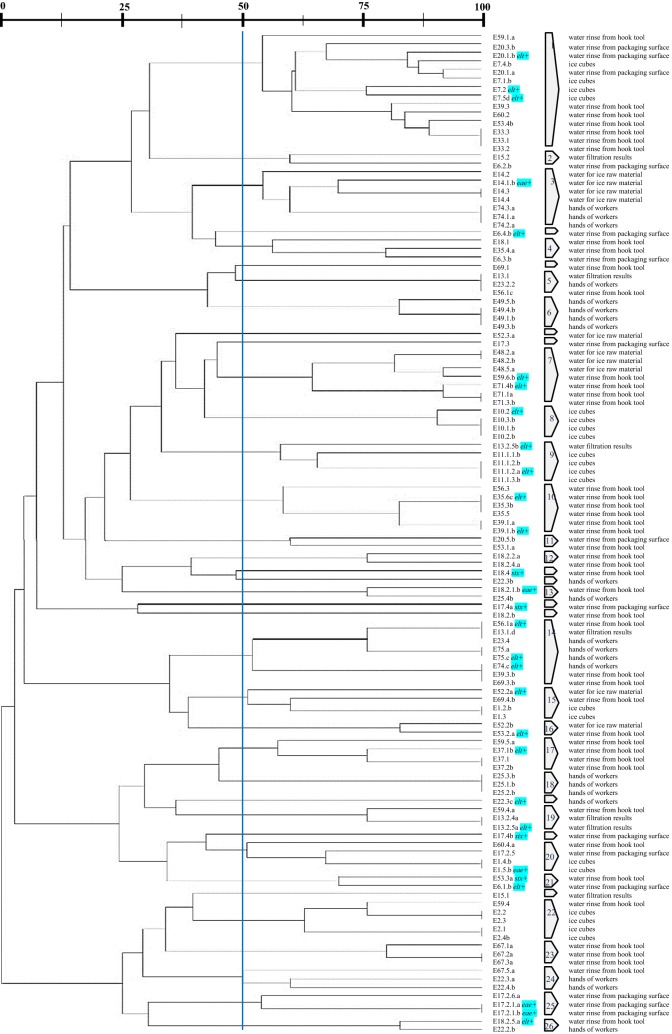
Dendrogram of *E. coli* isolates from ice cube manufacturers, derived from analysis ERIC-PCR profiles at 50% similarity level. Codes of isolates are directly opposite the source of isolation

**Fig. 3:**
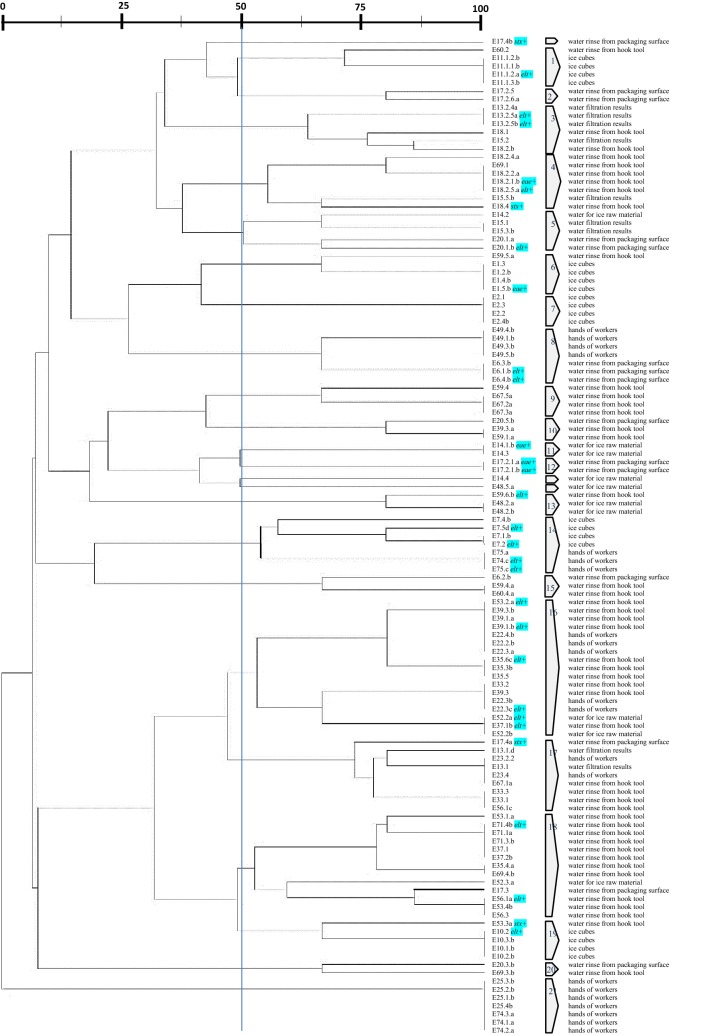
Dendrogram of *E. coli* isolates from ice cube manufacturers, derived from analysis REP-PCR profiles at 50% similarity level. Codes of isolates are directly opposite the source of isolation

ERIC and REP-PCR typing of 120 *E. coli* isolates tested at 50% similarity cut off value [17] were genetically diverse and consisted of heterogeneous population, all generated DNA patterns are relatively complex. Isolates in ERIC dendrogram were grouped into 26 clusters each containing at least two isolates (Fig. 2), whereas 11 fingerprints (9%) had individual lineages. Dendrogram for REP-PCR showed 21 clusters each of them containing at least two isolates with three fingerprints (2%) had individual lineages (Fig. 3).

Based on the source of *E. coli* isolates in ice cube manufacturers, isolates derived from one source of sampling sites in the ice cube manufacturer were not found to be in a group. High genetic diversity of *E. coli* were found in majority of sampling sites, such as *E. coli* isolates from ice cubes were found in many clusters i.e. 1, 8, 9, 15, 20 and 22. Then *E. coli* isolates from water rinse of worker’s hands were found in clusters 3, 5, 6, 14, 18, 24 and 26. *E. coli* isolates derived from water for ice raw material occurred in clusters 3, 7, 15 and 16. Next, isolates of *E. coli* from water filtration result were found in clusters 2, 5, 9, 14, and 19.

As is the result of the ERIC dendrogram, *E. coli* isolates on REP dendrogram also showed the same thing. *E. coli* isolates derived from the ice cubes as final product were found in clusters 6, 7, 14 and 19. Then isolates of *E. coli* from hands of worker were found in clusters 8, 14, 16, 17, 21. *E. coli* isolates derived from water for ice raw material were in clusters 5, 11, 13, 16 and 18. Isolates of *E. coli* from water filtration result were found in cluster 3, 4, 5, 17.

For site-based sample analysis, samples from city C were seen grouped in ERIC dendrogram on clusters 8, 15 and 20 and clusters 6 and 19 on the REP dendrogram. Cluster with a large number of isolates, as in ERIC dendrogram cluster 14 and REP dendrogram cluster 16, sample members can derived from samples of two cities, so it can be said that isolates are not clustered based on isolates origin cities.

From the previous study by Nikastrie, 2016, of the samples on major virulence genes associated with diarrheagenic *E. coli* (*eae*, *stx*, *elt*), 30 samples were positively detected positive virulence genes (5 samples positives *eae*, 4 samples positive *stx* and 21 samples positive *elt*). Dendrogram showed that the distribution of the genes were spread among clusters.

### Discussion

In this study, genetic variability of *E. coli* in populations from diverse habitat was analyzed using ERIC and REP-PCR fingerprinting since it has previously been shown to have a good discriminatory power as mentioned above. ERIC and REP-PCR DNA fingerprint analysis revealed extensive genetic diversity among *E. coli* strains even from the same sampling sites.

Equally found by Louws et al. [18], the total number of bands obtained by REP-PCR in this study were lower compared to ERIC PCR. This may rationalize how clusters generated with REP-PCR revealed less clusters than ERIC-PCR fingerprinting. Research done by Mohapatra et al. in [19, 20] showed that repetitive PCR method resulting more bands will create a larger number of cluster. Therefore we found that ERIC-PCR produce more discriminative fingerprint patterns than REP-PCR. When it comes to distinguishing between human and non-human isolates, Lipman et al. [21] observed that REP-PCR was less reliable than ERIC-PCR, whereas Leung et al. [22] found that ERIC-PCR was not an effective tool.

The presence of virulence genes in ice cube manufacturers is alarming, since there is possibility to be transferred to ice cubes. A lot of research has been done to determine *E. coli* contamination and their prevalence in water source [23–26] and drinking water [27–29]. Some studies on ice cube also indicated *E. coli* and pathogen contamination [30, 31]. Firlieyanti [2] showed that 45% ice cube samples in Bogor were fecal coliform positive and 10% sample were *E. coli* positive.

While this study presents that the most common virulence genes were *elt*, other study conducted in Canada reported that *eae* gene was the most frequently identified gene compared to the others in fecal material of various animal hosts [32]. High percentage of *est*II gene linked with intestinal pathogenic *E. coli* (IPEC) was found in sewage treatment plants and environmental waters in Queensland, Australia [33] whereas the most frequently detected of *E. coli* virulence genes in marine sediments in Rome, Italy were *traT* (involved in sepsis), *fyuA* and *ibeA* (involved in meningitis) [34]. This could be some fact that the abundance of virulence gene-carrying *E. coli* strains were differ depend on which environment or source or in other words pathogens that could be expected to occur in contaminated waters are dependent on the host source reservoir from which they are derived.

Water and low environmental temperatures as a source of contamination for *E. coli* has been noted by other study. In 1985, Dickens et al. [35] has revealed that Enteropathogenic bacteria including *E. coli* can survive when frozen in ice. Similarly, Falcão et al. [31] suggest that ice could be vehicle for enteric and other pathogens, while Kim et al. [36] showed clearer result that melting ice contaminated by *E. coli* could survive and transferred lettuce surfaces via melted ice. Dual regulation system which assist and maintain growth in water were suggested by Seurinck et al. [37] and the properties of peritrichous pili-flagella that aid movement in liquid environments that may enhance adaptation and survival [38]. The survival of *E. coli* follows an inverse relation with temperature. *E. coli* have a program in which low temperature causes a slowing down of the metabolism then cause a delay in cellular damage [39].

Virulence genes (*eae*, *stx*, *elt*) detected from isolates in this study were spread among the clusters in dendrogram. So, there was no correlation observed between phylogenetic relationship and virulence genes, whereas the isolates were not grouped according to the virulence factor pattern. With a lot of types virulence genes known in pathogenic *E. coli*, it is high possibility that other genes also occur in ice cube manufacturers. Here, we only focused on *eae*, *stx* and *elt* genes because they represent enteropathogenic *E. coli* (EPEC), enterohemorrhagic *E. coli* (EHEC) and enterotoxigenic *E. coli* (ETEC) respectively, which are included to be major causes of severe of diarrhea if it not treated properly [40].

As stated by Radu et al. [41], observing genetic diversity among the *E. coli* isolated by means of molecular typing techniques will contribute to the investigation of potential epidemiological problems caused by this pathogen, so that critical points can be identified and appropriate measures implemented to guarantee product safety.

In summary, we found that our samples indicated a variation in the occurrence of *E. coli* isolates in ice cube manufacturers, suggesting that they exhibit diverse population structures. ERIC-PCR method is more discriminative compared with REP-PCR to analyze the genetic diversity of *E. coli* from ice cubes production sites. This result can be used as a recommendation to the government as there is need for regular monitoring and counseling is required for producers, distributors and consumers of ice cubes.

## Limitations

Contamination of *E. coli* below limit detection cannot be detected in this research, though it may have contaminated the iced.

## Abbreviations

ERIC: enterobacterial repetitive intergenic consensus
REP-PCR: repetitive extragenic palindromic polymerase chain reaction
